# Adsorption, mobility, and degradation of the pesticide propaquizafop in five agricultural soils in China

**DOI:** 10.1038/s41598-023-32771-6

**Published:** 2023-04-10

**Authors:** Zhijia Cheng, Zhiguang Hou, Hongbo Han, Xiaolong Yu, Jiaxin Li, Qinghui Zhao, Ning Zhang, Zhongbin Lu

**Affiliations:** College of Plant Protection, Jilin Agricultural University, Changchun, 130118 China

**Keywords:** Environmental sciences, Environmental chemistry, Environmental impact

## Abstract

Propaquizafop is a fatty acid synthetic herbicide used to control annual and perennial grasses. To understand the potential environmental risks of propaquizafop to crops and food safety, the adsorption, mobility, and degradation of propaquizafop in five different soils were studied. At an initial concentration of 5 mg L^−1^ propaquizafop, its adsorption equilibrium was reached within 24 h, and the adsorption rates were between 46.98 and 57.76%. The Elovich kinetic model provided the best fit for the kinetic model, with *R*^2^ values between 0.9882 and 0.9940. For the isothermal adsorption tests, the Freundlich model was used to better fit the adsorption characteristics of propaquizafop in different soils, with *R*^2^ values between 0.9748 and 0.9885. Increasing the concentration of Ca^2+^ was beneficial for propaquizafop adsorption. In the soil thin-layer chromatography tests, the *R*_*f*_ of propaquizafop in the five soil samples ranged from 0.076 to 0.123. The results of the soil column leaching tests showed that propaquizafop did not migrate in the five soil columns; it was not detected in the leachate of each soil column, and propaquizafop in the soil columns only existed in the 0–5 cm soil layer. The results of soil thin-layer chromatography and soil column leaching tests showed that propaquizafop is a pesticide with a weak migration ability. Under the same environmental conditions, the degradation rate of propaquizafop in different soils followed the order LF fluvo–aquic soil (*T*_1/2_ = 1.41 d) > CS red loam (*T*_1/2_ = 2.76 d) > SX paddy soil (*T*_1/2_ = 3.52 d) > CC black soil (*T*_1/2_ = 5.74 d) > BS ginseng soil (*T*_1/2_ = 7.75 d). Considering the effects of soil moisture, incubation temperature, and microorganisms on propaquizafop degradation in the soil, temperature was found to have the greatest influence on its degradation rate.

## Introduction

Plant diseases, insect pests, and weeds are harmful to crops. According to the Food and Agriculture Organization of the United Nations (FAO), the annual loss of global food production owing to plant diseases, insect pests, and weeds is as high as 35%^1^. As an important means of control, pesticides can effectively reduce losses caused by diseases, pests, and weeds, and play a vital role in crop growth. Standardised use of pesticides can effectively control the occurrence of plant diseases, pests, and weeds, thereby improving crop yield and quality^2^. However, the increasing use of pesticides has caused great harm to the environment and is considered one of the major factors affecting environmental quality^3^. The soil is the ultimate destination for pesticides. Regardless of how the pesticide is applied, it enters the soil either directly or indirectly^4^. When a pesticide enters the soil, it undergoes a series of reactions, including adsorption, migration, and degradation, and causes long-term soil pollution^5^. The environmental behaviour of pesticides can determine their fate in the soil. Studies on the environmental behaviour of pesticides in soils have included processes such as leaching, degradation, bioaccumulation, adsorption, and desorption^6^. Studies on the environmental behaviour of pesticides can provide theoretical support for their harmful effects on the environment and groundwater.

**Figure 1:**
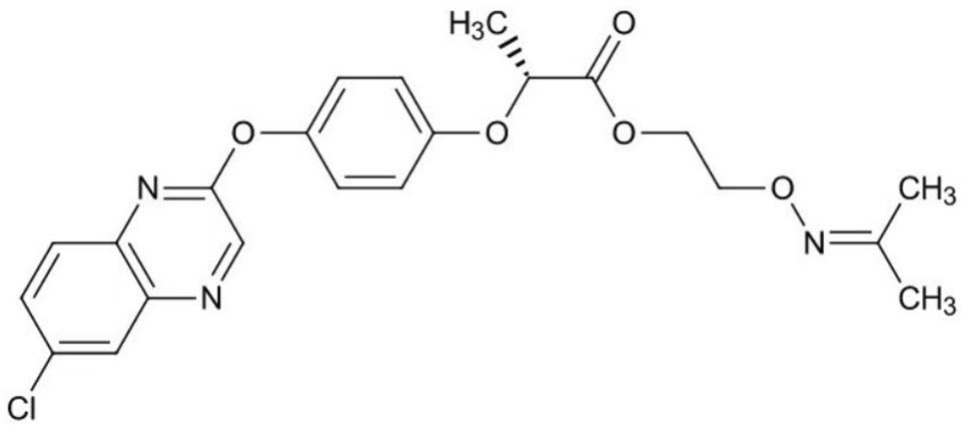
Chemical structure of propaquizafop.

Propaquizafop (IUPAC name 2-isopropylideneaminooxyethyl (R)-2-[4-(6-chloroquinoxalin-2-yloxy)phenoxy]propionate) is a fatty acid synthetic herbicide, whose chemical structure is shown in Fig. 1. The standard is a white powder with a solubility of 0.63 mg L^−1^ in water at 20 °C, 500,000 mg L^−1^ in acetone, 100,000 mg L^−1^ in chloroform, 76,000 mg L^−1^ in methanol, and 500,000 mg L^−1^ in toluene. At the temperature of 20 °C and pH 7, the octanol–water partition coefficient P is 6.03 × 104 (log P = 4.78), and the density is 0.96 g L^−1^, the vapour pressure at 20 °C is 4.39 × 10^–07^ mPa. The surface tension is 53.5–55.4 (mN m^−1^)^7^. After treatment with stems and leaves, propaquizafop was quickly absorbed by the leaves of grass weeds and transferred to the entire plant. It accumulates in plant meristems and inhibits the enzyme acetyl-CoA carboxylase, causing fatty acid synthesis that blocks and kills weeds. The pesticide was registered in China in 2018 as 10% propaquizafop EC and is mainly used to control annual or perennial gramineous weeds in crops such as rape, soybean, sunflower, and potato^8^. To date, studies on propaquizafop have mainly focused on the field-control effect of weeds and residue detection methods. Little research has been conducted on hydrolysis, photolysis, and soil degradation in terms of environmental behaviour^9–12^, and there are no reports on the adsorption behaviour of propaquizafop in soil. The adsorption properties of pesticides play a crucial role in the assessment of their behaviour and potential environmental risks. The adsorption and transport of herbicides in soil are major sources of drinking water and groundwater pollution in many countries^13^; thus, it is of great importance to study the environmental behaviour of herbicides in soil. In this study, the environmental behaviour of propaquizafop in five different soils was simulated in the laboratory, filling a gap in the study of the adsorption, migration, and degradation of propaquizafop in various soils. Baishan ginseng soil was selected as one of the tested soils because the author confirmed that the pesticide had a good control effect on grass weeds in ginseng fields and was safe for ginseng. The results of this study provide data to support the safety evaluation of propaquizafop in the environment and provide a theoretical basis for the registration of propaquizafop in ginseng.

## Materials and methods

### Reagents and materials

The propaquizafop standard (purity 99.8%) was purchased from Tianjin Alta Scientific. Chromatographically pure acetonitrile was purchased from MREDA Medical Technology Company. Pure FA, MgSO_4_, NaCl, and anhydrous CaCl_2_ were purchased from Xilong Science. Ultrapure water was obtained from a Milli-Q system (Millipore, Burlington, MA, USA).

Black soil was obtained from Changchun City, fluvo–aquic soil from Langfang City, red loam soil from Changsha City, paddy soil from Shaoxing City, and ginseng soil from Baishan City. Soil samples were randomly collected from the top layer of soil (0–20 cm), and the collected soil was dried naturally for two days at 25 °C indoors, after which the soil moisture evaporated. Then, the soil was manually ground and passed through a 2 mm screen. The physical and chemical properties of each soil sample are listed in Table 1.

**Table 1:** Physical and chemical properties of the five tested soil types.

Soil type	pH	OM (%)	CEC (cmol( +) kg^−1^)	Soil texture		
				Clay (< 2 µm) (%)	Silt (2–20 µm) (%)	Sand (> 20 µm) (%)
BS ginseng soil	6.20	4.6	20.38	21.28	44.86	33.96
SX paddy soil	6.10	3.79	17.0	29.2	67.6	3.2
CC black soil	7.43	3.36	27.3	39.2	41.8	19.0
LF fluvo–aquic soil	8.54	1.71	19.8	40.3	45.0	14.7
CS red loam	4.54	0.82	19.2	44.4	34.0	21.6

### Method for the determination of propaquizafop in CaCl_2_ solution and soil

To extract propaquizafop from water samples, a 1 mL sample was accurately transferred into a 5 mL polypropylene centrifuge tube, containing 2 mL acetonitrile and 500 µL 0.2% FA water. The mixture was vortexed for 2 min, followed by the addition of NaCl (1.0 g). The mixture was vortexed for 1 min and centrifuged at 5000 r min^−1^ for 5 min. The supernatant was passed through a 0.22 µm organic filter membrane using a sterile syringe and injected into vials for the determination of propaquizafop by high-performance liquid chromatography.

To extract propaquizafop from the soil samples, a soil sample (5–10 g) was accurately transferred into a 50 mL polypropylene centrifuge tube containing 10 mL acetonitrile and 2.5 mL 0.2% FA water. The mixture underwent 180 r min^−1^ oscillation for 0.5 h, followed by the addition of NaCl (2.0 g). The mixture was then vortexed for 1 min and centrifuged at 5000 r min^−1^ for 5 min. The supernatant was then passed through a 0.22 µm organic filter membrane using a sterile syringe and injected into vials for the determination of propaquizafop by high-performance liquid chromatography.

Liquid chromatography was performed under the following conditions: Agilent Technologies 1200 liquid chromatograph with Thermo BDS Hypersil C18 column (250 mm × 4.6 mm, 5 µm), mobile phase with water (A phase) and acetonitrile (B phase) (V: V/20:80), constant current of 1 mL min^−1^, column temperature of 30 °C, sample volume of 10 µm, UV detection wavelength of 230 nm, and retention time of 5.5 min.

The standard mother liquor of propaquizafop was added to a blank water sample and blank soil sample to carry out the addition recovery rate test; the standard addition levels were 0.1, 1, 10 mg L^−1^ (water sample) and 1, 5, 10 µg g^−1^ (soil sample). The recovery of propaquizafop from the water samples was 87.91–107.61% with a relative standard deviation of 1.58–5.23%. The recovery of propaquizafop from the soil samples was 87.56–103.56% with a relative standard deviation of 1.83–6.37%.

### Adsorption

#### Soil-to-solution ratio in sorption and desorption

According to the method described by OECD106^14^, the optimum soil-solution ratio for the adsorption test was determined using the oscillatory equilibrium method. First, a solution containing 0.01 mol L^−1^ CaCl_2_ and 0.01 mol L^−1^ NaN_3_ was prepared. The concentration of propaquizafop in the solution was set to 5 mg L^−1^ by adding 0.25 mL 1000 mg L^−1^ propaquizafop standard mother liquor prepared with acetonitrile. Acetonitrile had no effect on adsorption, and its mass fraction in the solution was 0.5%. CaCl_2_ in solution was used to simulate the background value of the electrolyte in the soil solution, and NaN_3_ was used to inhibit microbial activity and prevent microbial degradation of the pesticide. Because of the low solubility of propaquizafop in water, 1% polysorbate 80 was added to the solution to make propaquizafop more soluble. The optimal soil-solution ratio was determined by setting the soil-solution ratio, 1:5 (1 g soil, 5 mL of 0.01 mol L^−1^ CaCl_2_ and 0.01 mol L^−1^ NaN_3_ solution), 1:10 (1 g soil, 10 mL of 0.01 mol L^−1^ CaCl_2_ and 0.01 mol L^−1^ NaN_3_ solution), 1:20 (1 g soil, 20 mL of 0.01 mol L^−1^ CaCl_2_ and 0.01 mol L^−1^ NaN_3_ solution), and 1:50 (1 g soil, 50 mL of 0.01 mol L^−1^ CaCl_2_ and 0.01 mol L^−1^ NaN_3_ solution). Five kinds of soil samples were collected, and the quantitative soil samples were mixed with 0.01 mol L^−1^ CaCl_2_ and 0.01 mol L^−1^ NaN_3_ solution in a 250 mL conical flask, which was then placed in a constant temperature oscillator with a speed of 150 r min^−1^ at 25 °C for 24 h, and centrifuged in a 5000 r min^−1^ for 10 min. The supernatant was collected to determine propaquizafop concentration in the solution. Simultaneously, propaquizafop was added without soil treatment as a blank control, and all treatments were repeated thrice.

#### Adsorption kinetics

The kinetic data of propaquizafop adsorption in different soils were determined using the batch oscillatory equilibrium method^14^. One gram of soil was mixed with 50 mL of 1% polysorbate 80 and 0.01 mol L^−1^ CaCl_2_ and 0.01 mol L^−1^ NaN_3_ solution in a 250 mL conical flask. Then, 0.25 mL of 1000 mg L^−1^ propaquizafop standard mother liquor prepared with acetonitrile was added into the conical bottle, with a final concentration of propaquizafop of 5 mg L^−1^. The samples were mixed in a constant temperature oscillator with a speed of 150 r min^−1^ at a temperature of 25 °C and sampled at 0, 1, 3, 6, 10, 16, 24, and 48 h of oscillation, respectively, and finally centrifuged for 10 min at 5000 r min^−1^. The concentration of propaquizafop in the supernatant was determined, and all the treatments were repeated thrice.

#### Adsorption isotherms

The isothermal adsorption of propaquizafop in five types of soil was studied using the oscillatory equilibrium method^14^. A volume of 50 mL of 1% polysorbate 80 and 0.01 mol L^−1^ CaCl_2_ and 0.01 mol L^−1^ NaN_3_ solution were mixed and then the standard solution of propaquizafop prepared with acetonitrile was added. The concentrations of propaquizafop in the conical flask were 1, 5, 10, 15, 20, and 25 mg L^−1^. The conical flask was transferred to a thermostatic oscillator and was oscillated at 150 r min^−1^ for 24 h at 25 °C. After centrifugation for 10 min at 5000 r min^−1^, the concentration of propaquizafop in the supernatant was determined. All processing settings were repeated thrice.

#### Effects of Ca^2+^ concentration on adsorption

The effects of Ca^2+^ concentration on the adsorption of propaquizafop in different soils were studied using the oscillatory equilibrium method^14^. CaCl_2_ was used as a solvent to prepare different concentrations of Ca^2+^ background solutions (0.1, 0.3, 0.5, 0.7, and 1 mol L^−1^) to investigate the effects of Ca^2+^ concentration on adsorption. The concentration of propaquizafop in the solution was 5 mg L^−1^, and 1% polysorbate 80 was added as a cosolvent. All treatments were repeated thrice.

### Thin-layer chromatography of soil

According to the “GB/T 31270.5-2014”^15^, a 10 g sample of air-dried and sieved soil (0.25 mm) was mixed in a 50 mL beaker with 7 mL distilled water until sludgy^16,17^, and a glass rod was used to evenly smear the soil slurry onto a 20 cm × 7 cm glass plate. The thickness of the soil layer was controlled between 0.5 and 1 mm^18,19^. At room temperature (25 °C), the coated glass plate was dried naturally in the dark, 10 µL of 1000 mg L^−1^ propaquizafop standard solution was added 1.5 cm away from the bottom of the plate, and the sheet was placed in a tank containing pure water (water level 0.5 cm) at an inclined angle of approximately 30°. The sheet was removed when it reached a height of 2 cm from the top and dried naturally in the dark at room temperature. After drying, the soil on the plate was divided into five equal parts (0–3, 3–6, 6–9, 9–12, 12–15, and 15–18 cm)^19,20^. The propaquizafop content in each part of the soil was determined using chromatography.

### Leaching of soil

The air-dried and screened soil (0.25 mm) weighed 600–700 g and was placed into a plastic pipe (5 cm inner diameter and 32 cm height) to make a 30 cm high soil column. The soil column was saturated to equilibrium by reverse osmosis with a 0.01 mol L^−1^ CaCl_2_ solution. A standard 1 mL solution of 1000 mg L^−1^ propaquizafop was evenly dripped onto the top surface of the soil column. After the acetonitrile evaporated completely, quartz sand with a thickness of 1 cm was added to the top of the soil column. 0.01 mol L^−1^ CaCl_2_ solution was used as the eluent, and 300 mL of the eluent was completely eluted within 14.5 h. The collected eluent was pretreated using the extraction method described in “Method for the determination of propaquizafop in CaCl2 solution and soil” section, and the propaquizafop concentration was analysed by high-performance liquid chromatography. The soil in the column was divided evenly into six parts. The propaquizafop contents in different parts of the soil were determined after natural drying. Each process was repeated three times^21,22^.

### Degradation of propaquizafop in different types of soil

Each soil sample (5 g) was placed in a 50 mL polypropylene centrifuge tube (10 sampling time points were set for each soil sample), and 0.25 mL of 100 mg L^−1^ propaquizafop standard solution was added. The concentration of propaquizafop in the soil was 5 µg g^−1^, and when the solvent was volatilised completely, the soil moisture was adjusted to 60% of the field maximum water holding capacity by adding sterilised distilled water. The soil samples were then placed in a constant temperature incubator at 25 ± 1 °C to be cultured in the dark, weighed regularly, and replenished with water to ensure the soil moisture content. Samples were collected regularly after the start of the experiment to determine the propaquizafop concentration in the soil. All experiments were performed in triplicates.

### Degradation of propaquizafop in BS ginseng soil under different conditions

The soil was sterilised as follows: the soil was autoclaved for 30 min in a sterilisation oven at 120 °C. The soil was incubated in a thermostat for 6 h and then sterilised again. This step was repeated thrice.

Soil without organic matter was obtained as follows: a certain amount of soil in the beaker was placed in a 70 °C water bath. Hydrogen peroxide (30%) was added, and the mixture was stirred continuously until the reaction was complete. The soil was washed several times with deionised water. After natural air-drying of the soil, the soil sample was sifted through a 2 mm sieve.

The degradation dynamics of propaquizafop in sterilised and organics-free BS ginseng soils were studied. The content of propaquizafop in soil was 5 µg g^−1^, and the soil water content was 60% of the field maximum water holding capacity at 25 ± 1 °C. The propaquizafop content in ginseng soil was regularly detected.

To understand the effect of different temperatures on the degradation of propaquizafop in BS ginseng soil, the temperature conditions of 25 °C, 35 °C, and 50 °C were set to sterilised and unsterilised ginseng soil without light. The content of propaquizafop in the soil was 5 µg g^−1^, the soil moisture content was 60% of the maximum field water holding capacity, and the content of propaquizafop in ginseng soil at the different temperatures was detected regularly.

The effect of the soil water content on the degradation of propaquizafop in ginseng soil was studied by setting the soil water content to 20%, 60%, and 100% of the field’s maximum water-holding capacity. The content of propaquizafop in soil was 5 µg g^−1^ at 25 ± 1 °C. The propaquizafop content of the ginseng soil was measured periodically.

### Data analysis

In this study, we used pseudo-first-order, pseudo-second-order, Elovich, and Weber–Morris intraparticle diffusion adsorption kinetics models to analyse the adsorption characteristics of propaquizafop.

$${q}_{t}={C}_{e}\times \left(1-{e}^{-{k}_{1}t}\right),$$

$${q}_{t}=\frac{{k}_{2}{q}_{e}^{2}t}{1+{{k}_{2}q}_{e}t},$$

$${q}_{t}=\frac{1}{\beta }{\text{ln}}\left(\alpha \beta \right)+\frac{1}{\beta }{\text{ln}}\left(t\right),$$

$${q}_{t}=kw\times {t}^{0.5}+c,$$

where *q*_*t*_ and *q*_*e*_ (mg kg^−1^) are the adsorption capacities of propaquizafop in the soil at time *t* and equilibrium, respectively. The variables *k*1, *k*2*,* and *k* are the first-order, pseudo-second-order, and intraparticle diffusion rate constants, respectively. Parameter *α* is the initial chemical adsorption rate, and *β* is the adsorption–desorption constant. *C* is the relative thickness of the boundary layer.

The relationship between propaquizafop concentration in the solution and propaquizafop adsorption in the soil at adsorption equilibrium was described using the Freundlich and Langmuir models as follows:

$${C}_{s}={K}_{F}{\cdot C}_{e}^\frac{1}{n},$$

$${C}_{s}={q}_{max}\cdot \frac{{K}_{L}{C}_{e}}{{1+K}_{L}{C}_{e}},$$

where *C*_*s*_ (mg kg^−1^) is the equilibrium adsorption capacity of propaquizafop in the soil; *C*_*e*_ (mg L^−1^) is the equilibrium adsorption concentration of propaquizafop in the solution; and *K*_*F*_ (L kg^−1^) and K_L_ (L kg^−1^) are the adsorption constants of the Freundlich and Langmuir models, respectively, which are related to the propaquizafop adsorption capacity and intensity.

The adsorption constants for organic carbon (*K*_*OC*_), Gibbs free energy change of adsorption (∆*G*), and ground ubiquity score (*GUS*) are as follows:

$$OC\%=OM\%/1.724,$$

$${K}_{oc}=100\times \frac{{K}_{F}}{OC\%},$$

$$\Delta G=-RT{\text{ln}}\left({K}_{F}\right),$$

$$GUS={\text{lg}}{T}_{1/2}\times \left(4-{\text{lg}}{K}_{oc}\right),$$

where *OC%* and *OM*% are constants for organic carbon and organic matter, respectively, *R* is the gas constant [8.314 J/(K mol)], and *T* is the absolute temperature.

The mobility factor (*R*_*f*_) of the thin-layer soil plates was calculated as follows:

$${R}_{f}=\frac{\Sigma {Z}_{i}\times {M}_{i}}{{Z}_{W}\times \Sigma {M}_{i}},$$

where *Z*_*w*_ is the distance moved by the developing solvent from the starting point (cm); *i* is the number of segments; *Z*_*i*_ is the distance of segment *i* from the starting point (cm); and *M*_*i*_ is the pesticide content of segment *i* (mg).

The first-order kinetic model was used to fit the propaquizafop degradation kinetics and was calculated as follows:

$${C}_{t}{=C}_{0}{e}^{-kt},$$

$${T}_{1/2}=\frac{\ln 2}{k},$$

where *t* is the time (d or h), *C*_*t*_ is the concentration of propaquizafop residue in the soil at time *t* (mg kg^−1^), *C*_0_ is the initial concentration of propaquizafop when added to the soil (mg kg^−1^), *k* is the first-order kinetic constant (d^−1^), and *t*_1/2_ is the half-life of propaquizafop degradation in the soil (d).

## Results and discussion

### Propaquizafop adsorption in soil

#### Effects of soil-to-solution ratios on propaquizafop adsorption

We determined the effects of different soil-to-solution ratios on propaquizafop adsorption in different soils (Table 2). The results showed that, under different soil-to-solution ratios, the adsorption rates of the five soils for propaquizafop were all high, ranging from 50.68 to 93.80%, and decreased with an increase in the soil-to-solution ratio. The soil-to-solution ratio requires that the pesticide adsorption rate in the soil be more than 50% so that a stable soil suspension can be maintained during the oscillation process. It can be seen that the adsorption rate of the four treatments was more than 50%; all met the requirements. In addition, according to the requirements of GB/T 31270.4-2014 for adsorption tests^23^, the soil-to-solution ratio should be high when the solubility of the samples is low. Therefore, the soil-to-solution ratio for the adsorption tests of the five soil types was determined to be 1:50.

**Table 2:** Adsorption rate of propaquizafop in different soils under different soil-to-solution ratios.

Soil type	Soil-to-solution ratio	Equilibrium concentration (mg L^−1^)	Equilibrium adsorption capacity (mg g^−1^)	Adsorption ratio (%)
BS ginseng soil	1:5	0.31	234.50	93.80
	1:10	0.97	201.70	80.68
	1:20	1.78	160.80	64.32
	1:50	1.97	151.70	60.68
SX paddy soil	1:5	0.37	231.60	92.64
	1:10	1.00	199.90	79.96
	1:20	1.48	176.10	70.44
	1:50	2.12	144.20	57.68
CC black soil	1:5	0.59	220.40	88.16
	1:10	1.27	186.50	74.60
	1:20	1.84	157.90	63.16
	1:50	2.16	141.80	56.72
LF fluvo–aquic soil	1:5	0.77	211.50	84.60
	1:10	1.49	175.50	70.20
	1:20	2.13	143.40	57.36
	1:50	2.47	126.70	50.68
CS red loam	1:5	1.15	192.30	76.92
	1:10	1.70	165.20	66.08
	1:20	1.96	151.90	60.76
	1:50	2.39	130.50	52.20

### Adsorption kinetics of propaquizafop in soil

**Figure 2:**
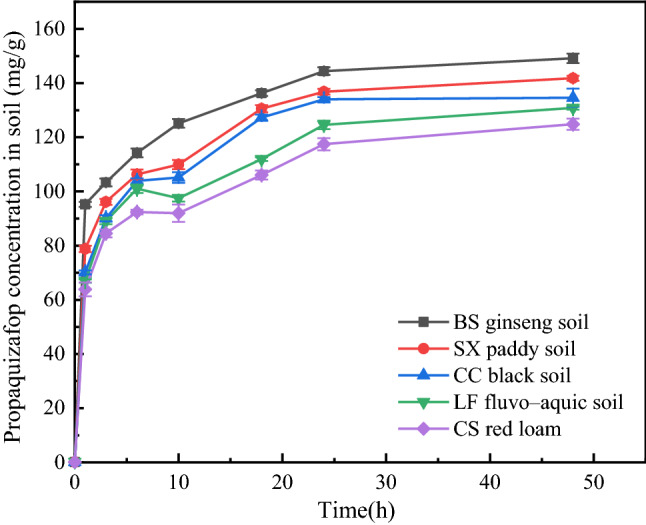
Adsorption kinetics of propaquizafop in different soil types (n = 3).

The adsorption of pesticides in the soil is a dynamic equilibrium process, and the adsorption of propaquizafop in different soils over time is shown in Fig. 2. It can be seen that the adsorption of propaquizafop in different soils can be divided into three stages. In the first stage, propaquizafop was rapidly adsorbed onto each soil within 6 h of the beginning of adsorption. The adsorption rate was faster, and the adsorption amount increased rapidly, possibly because of physical adsorption on the soil surface and the chemical and hydrogen bonds between propaquizafop and minerals in the soil. A higher concentration of propaquizafop in solution leads to a higher molecular collision frequency, which can cause propaquizafop to rapidly adsorb onto the surface of soil particles in the state of dissolution. In the second stage, the adsorption rate decreased significantly between 6 and 10 h owing to the larger amount of propaquizafop adsorbed by the soil in the early stage of adsorption. This led to a decrease in the concentration gradient of propaquizafop in the solution, causing the adsorption rate to gradually decrease. After the third stage (10 h), the adsorption capacity of each soil increased to a low degree, until the adsorption process lasted 24 h, at which time the adsorption had reached an equilibrium state, indicating that the adsorption process was complete.

The adsorption kinetics model was used to describe the rate equation of liquid–solid adsorption kinetics. The rate constants and orders of the reactions were obtained by fitting the kinetic models relative to the experimental data. The kinetic model described the growth characteristics of the reaction rate over time during the entire adsorption process^24^. Four kinetic models were used to fit the adsorption kinetics of propaquizafop in different soils, and the parameters of the four kinetic models are shown in Table 3. Five soils were better fitted by the Elovich kinetics equation, with* R*^2^ values between 0.9882 and 0.9940. Some studies have shown that the Elovich model is more suitable for studying the adsorption kinetics in sediments or soils with large variations in activation energy^24^. Furthermore, the adsorption energies of the five soils changed significantly during adsorption. In addition, the Elovich dynamics model can reveal heterogeneous diffusion processes, which other models ignore.

**Table 3:** Constants and correlation coefficients for the kinetics models.

Kinetics models	Parameters	BS ginseng soil	SX paddy soil	CC black soil	LF fluvo–aquic soil	CS red loam
Pseudo-first-order	*k*_*1*_	1.1094	0.7748	0.6081	0.7150	0.7325
	*q*_*e*_	130.5771	123.8439	121.1875	112.6495	106.0375
	*R*^2^	0.8955	0.8897	0.9009	0.9039	0.8918
Pseudo-second-order	*k*_2_	2.2414E45	1.55739E46	3.21224E44	− 6.16609E34 ±	− 3.23464E45
	*q*_*e*_	123.9647	114.3336	109.2904	103.1201	97.3052
	*R*^2^	0.8136	0.7445	0.7052	0.7256	0.7226
Elovich	*α*	5620.7328	1507.7674	900.0522	1097.2619	983.2959
	*β*	0.0650	0.0580	0.0556	0.0619	0.0649
	*R*^2^	0.9940	0.9925	0.9882	0.9882	0.9895
Weber–Morris	*k*_*w*_	56.0510	54.4605	53.2875	49.6458	47.0740
	*C*	20.4667	14.5272	11.9789	12.2766	11.2274
	*R*^2^	0.9078	0.9422	0.9447	0.9430	0.9503

#### Adsorption of propaquizafop at equilibrium

**Figure 3:**
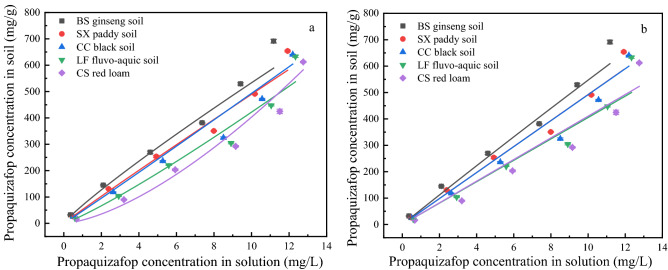
(**a**) Freundlich and (**b**) Langmuir modes isotherms of propaquizafop in five different agricultural soils (n = 3).

The adsorption of pesticides on soil is mainly based on distribution theory and adsorption point theory^25^. The interactions between the soil and pesticides are complex and include ion exchange, complexation, and chemical bonding. The isothermal adsorption curves of propaquizafop in the five soil types are shown in Fig. 3. The Freundlich and Langmuir isothermal adsorption models were used to describe the sorption isotherms of propaquizafop in the five soils. The parameters of these two models are listed in Table 4. It can be seen that the average *R*^2^ value of the Freundlich model is more suitable than that of the Langmuir model. This was further illustrated using the Freundlich model, which could be used to better fit the adsorption characteristics of propaquizafop in different soils. In the Freundlich model, *K*_*F*_ represents the adsorption constant of the adsorption intensity of the soil, and *n* is a parameter related to the adsorption intensity of the adsorbent molecules. The *K*_*F*_ of propaquizafop in the five soils was between 12.3440 and 68.3757, and the difference in the adsorption strength of propaquizafop in the different soils may be related to their physical and chemical properties. For the Freundlich model, *1/n* > 1 represents unfavourable adsorption conditions and *1/n* < 1 represents favourable adsorption conditions. The values of *1/n* are between 0.8921 and 1.5136. The results showed favourable adsorption conditions in BS ginseng soil and SX paddy soil, and unfavourable adsorption conditions in CC black soil, LF fluvo–aquic soil, and CS red loam.

**Table 4:** Fitting results of the Freundlich and Langmuir model equations for the isothermal adsorption of propaquizafop in five different agricultural soils.

Soil	Freundlich model			Langmuir model		
	*K*_*F*_ (mg^1–n^/L·mg)	*1/n*	*R*^2^	*q*_*m*_ (mg/g)	*K*_*L*_ (L/mg)	*R*^2^
BS ginseng soil	68.3757	0.8921	0.9862	18,776.3097	0.0030	0.9828
SX paddy soil	52.3827	0.9706	0.9855	1.5324E6	3.2137E−5	0.9851
CC black soil	46.8722	1.0221	0.9885	7.2854E6	6.7505E−6	0.9881
LF fluvo–aquic soil	30.0191	1.1477	0.9748	8.4176E6	4.8129E−6	0.9659
CS red loam	12.3440	1.5136	0.9796	1.8950E7	2.1650E−6	0.9389

The adsorption of pesticides in soil is closely related to its organic matter content; therefore, the adsorption coefficient of organic carbon (*K*_*oc*_) can be used to evaluate the adsorption of pesticides in soil. According to the classification of pesticide adsorption characteristics in the soil adsorption and desorption test criteria (GB/T 31270.4-2014)^23^, when 1000 < *K*_*oc*_ ≤ 5000, the pesticide has medium soil adsorption. As shown in Table 5, the* K*_*OC*_ values of propaquizafop in different soils were between 1000 and 5000, indicating that propaquizafop is a medium soil adsorption pesticide in different soils. Therefore, runoff does not easily occur on ginseng slopes in Northeast China. It is safe and the risk of nearby water pollution is minimal. The ∆*G* values of propaquizafop in soil ranged from − 6.23 to − 10.74 kJ mol^−1^ (298.15 K); these values were all less than 40 kJ mol^−1^, indicating physical adsorption of propaquizafop in soil. The groundwater generality score (*GUS*) was used to measure the leaching potential of chemicals and estimate the risk of pollutants in groundwater^26^. The *GUS* values of propaquizafop in BS ginseng soil, SX paddy soil, CC black soil, LF fluvo–aquic soil, and CS red loam were 0.53, 0.34, 0.47, 0.08, and 0.26, respectively, and much lower than 1.8. Typically, pesticides with a *GUS* higher than 2.8 are considered leachers, whereas pesticides with a *GUS* lower than 1.8 are regarded as non-leachers^27,28^. Our results suggest that propaquizafop has low leaching potential in soils.

**Table 5:** Adsorption constants, Gibbs free energy, and ground ubiquity score (*GUS*) of propaquizafop in five different agricultural soils (298.15 K).

Soil	*K*_*OC*_	∆*G* (kJ/mol)	*GUS*
BS ginseng soil	2562.60	− 10.47	0.53
SX paddy soil	2382.79	− 9.81	0.34
CC black soil	2404.99	− 9.53	0.47
LF fluvo–aquic soil	3026.49	− 8.43	0.08
CS red loam	2595.25	− 6.23	0.26

#### Effect of Ca^2+^ concentration of adsorption background solution on adsorption

**Figure 4:**
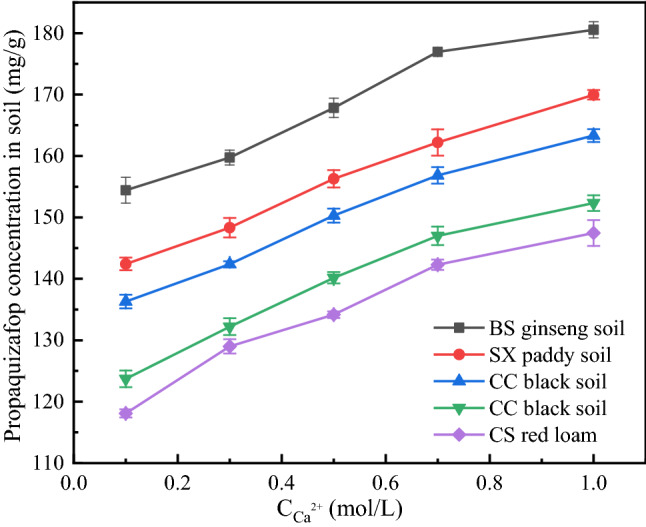
Effects of Ca^2+^ on the adsorption of propaquizafop (n = 3).

To understand the effects of ionic strength on the adsorption of propaquizafop in different soils, propaquizafop solutions with different concentrations of Ca^2+^ were prepared, with CaCl_2_, at 0.1, 0.3, 0.5, 0.7, and 1 mol L^−1^. The effects of background solutions with different Ca^2+^ concentrations on propaquizafop adsorption in the five soils are shown in Fig. 4. The adsorption of propaquizafop in the five soils increased gradually with the Ca^2+^ concentration. When the concentration of Ca^2+^ in the solution was 1 mol L^−1^, the adsorption of propaquizafop onto the five soils reached its peak value. This may be because the electrostatic interaction between the adsorptive and the soil is weakened and the hydrophobic interaction is enhanced when the ionic strength of the solution is increased. In addition, because Ca^2+^ and propaquizafop compete for solvent molecules, the solubility and salting-out of propaquizafop decreases, and the adsorption capacity of propaquizafop in the soil increases.

### Propaquizafop transport in soils

**Figure 5:**
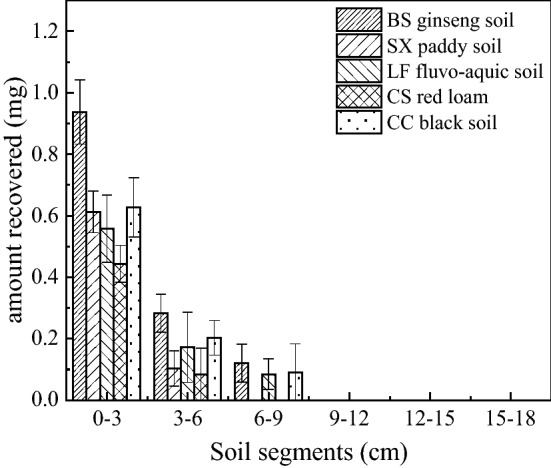
Distribution of propaquizafop in different soil plates.

Thin-layer chromatography and column leaching were used to study the vertical migration of propaquizafop in different soils. The results of thin-layer chromatography showed that the propaquizafop content in the first section of each thin layer was the highest, and 0.076 < *R*_*f*_ < 0.10, indicating that propaquizafop is a non-mobile pesticide in the SX paddy soil and BS ginseng soil. In the CS red soil, LF fluvo–aquic soil, and CC black soil, propaquizafop was not a mobile pesticide (Table 6). However, its distribution varied slightly from soil to soil, in which the distribution of propaquizafop in BS ginseng soil, CC black soil, and LF fluvo–aquic soil was in the thin layers of the first, second, and third stages of soil, whereas that in SX paddy soil and laterite was in the thin layers of the first and second stages of soil. This may have resulted from the different physical and chemical properties of the five soils, which affected the adsorption intensity of propaquizafop in each soil (Fig. 5). In the soil column leaching test, propaquizafop did not migrate in the five soil columns, was not detected in the leachate of each soil column, and was distributed in the soil column only in the 0–5 cm soil layer (Table 7).

**Table 6:** Values of propaquizafop in five soil plates (n = 3).

Soil	SX paddy soil	BS ginseng soil	CS red loam	LF fluvo–aquic soil	CC black soil
*R*_*f*_	0.076 ± 0.015	0.097 ± 0.012	0.101 ± 0.010	0.112 ± 0.010	0.123 ± 0.045

**Table 7:** Distribution of propaquizafop in the column filled with different soils.

Soil	Pesticide content in each section of the soil column (mg)						Cumulative content of propaquizafop in leachate (mg)
	0–5 cm	5–10 cm	10–15 cm	15–20 cm	20–25 cm	25–30 cm	
BS ginseng soil	2.19	0	0	0	0	0	ND
SX paddy soil	2.21	0	0	0	0	0	ND
LF fluvo–aquic soil	1.15	0	0	0	0	0	ND
CS red loam	1.09	0	0	0	0	0	ND
CC black soil	1.27	0	0	0	0	0	ND

In general, the mobility of propaquizafop in soil was weak, which was consistent with the higher adsorption rate observed in the adsorption experiment. This weak mobility may result from the low solubility of propaquizafop in water, which makes it less likely to enter the aqueous phase when water is distributed in the soil.

### Propaquizafop degradation in soils

#### Degradation of propaquizafop in different soil types

There are many factors affecting the degradation of pesticides in soil. To determine the effects of soil types on the degradation of propaquizafop, five typical agricultural soils were selected. The degradation of propaquizafop in different soils followed the first-order kinetics model, with *R*^2^ between 0.91 and 0.96, and the half-lives in the different soils were ginseng soil (7.75 d) > black soil (5.74 d) > paddy soil (3.52 d) > red loam (2.76 d) > fluvo–aquic soil (1.41 d) (Table 8), according to the classification of pesticide degradability in the soil (Test guidelines on environmental safety assessment for chemical pesticides-Part 4: Transformation in soils)^29^. Propaquizafop is a readily degradable pesticide in different soils (*t*_0.5_ < 30 d); however, its degradation rate in different soils varies, which may be related to the soil’s physical and chemical properties and soil microbial genera.

**Table 8:** Degradation kinetics parameters of propaquizafop in different soils.

Soil	Degradation kinetics equation	*k* (d^−1^)	*R*^2^	*T*_1/2_ (d)
BS ginseng soil	C = 2.3278e^−0.08945t^	0.08945	0.91699	7.75
SX paddy soil	C = 2.3620e^−0.19672t^	0.19672	0.92255	3.52
LF fluvo–aquic soil	C = 2.3168e^−0.49215t^	0.49215	0.96364	1.41
CS red loam	C = 2.2080e^−0.25075t^	0.25075	0.96105	2.76
CC black soil	C = 2.2505e^−0.12085t^	0.12085	0.92716	5.74

#### Effects of different conditions on the degradation of propaquizafop in BS ginseng soil

**Figure 6:**
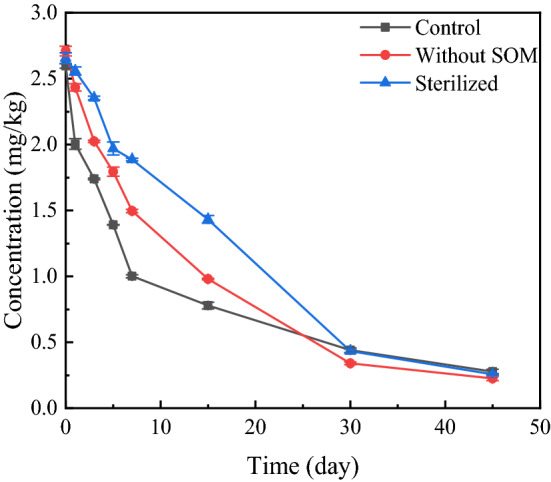
Degradation of propaquizafop in sterilised and organic matter-removed soil.

Soil microbial and organic matter contents had a greater impact on pesticide degradation in the soil. We sterilised the soil and removed organic matter to clarify its effect on the degradation of propaquizafop in BS ginseng soil. The degradation of propaquizafop in soil treated by sterilised and organic matter removal followed the first-order kinetics model, with* R*^2^ values of 0.98 and 0.99 and half-lives of 13.62 d and 9.83 d, respectively. The half-life of propaquizafop in ginseng soil was prolonged (t_0.5_ > 7.75 d) after sterilisation and organic matter treatment (Table 9, Fig. 6). However, *t*_0.5_ was still less than 30 d, and propaquizafop was still an easily degradable pesticide in both sterilised soil and soil with organic matter removal.

**Table 9:** Degradation kinetics parameters of propaquizafop in non-SOM and sterilised ginseng soils.

Treatment	Degradation kinetics equation	*k*	*R*^2^	*T*_1/2_ (d)
control	C = 2.32777e^−0.08945t^	0.08945	0.91699	7.75
Without SOM	C = 2.60329e^−0.07051t^	0.07051	0.99054	9.83
Sterilised	C = 2.68192e^−0.05091t^	0.05091	0.98604	13.62

**Figure 7:**
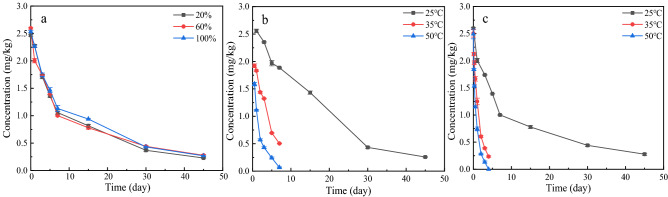
Propaquizafop degradation in ginseng soil under different conditions ((**a**) different moisture content; (**b**) sterilised soil at different temperatures; (**c**) unsterilised soil at different temperatures).

Microbial reproduction in the soil is closely related to the soil water content; therefore, the degradation of propaquizafop in the soil may be caused by both chemical hydrolysis and microbial activity. Under the condition of avoiding light at 25 °C, when the soil water content was 20%, 60%, and 100% of the saturated water content, the degradation of propaquizafop in ginseng soil followed the first-order kinetics model. Here, *R*^2^ values were 0.94, 0.91, and 0.93, respectively, and the half-lives were 7.72 d, 7.75 d, and 9.14 d, respectively (Table 10, Fig. 7a). Because of the low solubility of propaquizafop in water, propaquizafop in the water and soil phases enters the soil first; thus, water does not affect its distribution. At both low (20%) and high (60%) water content, propaquizafop penetrated the soil almost entirely. The half-life of propaquizafop did not change significantly between cases, which may be related to the microbial genera and soil content. When the soil water capacity reaches saturation, the soil–water system is anoxic and almost waterlogged, and the activity of aerobic microorganisms is inhibited, resulting in a slower degradation rate and longer half-life^30^.

**Table 10:** Degradation kinetics parameters of propaquizafop in BS ginseng soil under different conditions.

Condition	Treatment	Degradation kinetics equation	*k*	*R*^2^	*T*_1/2_ (d)
Soil moisture	20%	C = 2.36482e^−0.08983t^	0.08983	0.9476	7.72
	60%	C = 2.32777e^−0.08945t^	0.08945	0.91699	7.75
	100%	C = 2.35124 e^−0.07582t^	0.07582	0.93799	9.14
Temperature	25 °C	C = 2.32777e^−0.08945t^	0.08945	0.91699	7.75
	35 °C	C = 2.37909e^−0.65939t^	0.65939	0.99209	1.05
	50 °C	C = 2.30497e^−1.29351t^	1.29351	0.97842	0.54
Temperature sterilised	25 °C	C = 2.68192e^−0.05091t^	0.05091	0.98604	13.62
	35 °C	C = 2.33132e^−0.22583t^	0.22583	0.97436	3.07
	50 °C	C = 2.38943e^−0.70983t^	0.70983	0.98137	0.98

The effects of different temperatures on propaquizafop degradation in sterilised and non-sterilised ginseng soils were studied. The degradation of propaquizafop at 25 °C, 35 °C, and 50 °C in both sterilised and non-sterilised soils followed the first-order kinetics model. In the unsterilised soil, the* R*^2^ values were 0.91, 0.99, and 0.97, and the half-lives were 7.75 d, 1.05 d, and 0.54 d (Table 10, Fig. 7b), respectively. The degradation rate of propaquizafop in the unsterilised ginseng soil increased rapidly with increasing temperature. In sterilised soil, the degradation rate of propaquizafop was similar to that in unsterilised soil, but with a slower degradation rate. In the sterilised soil at the different temperatures, the *R*^2^ values were 0.98, 0.97, and 0.98, and the half-lives were 13.62 d, 3.07 d, and 0.98 d (Table 10, Fig. 7c), respectively. Although the degradation of propaquizafop in ginseng soil is greatly affected by temperature, microbes also have an effect under these conditions. Therefore, we conclude that there are two main reasons for the increase in the degradation rate that accompanies an increase in temperature: the increase in temperature itself, and the temperature range that facilitates the growth and reproduction of soil microorganisms. Increased microbial activity and abundance increase enzyme activity in the soil and, ultimately, the rate of degradation of propaquizafop in ginseng soil^31^. However, the effect of microorganisms on the degradation of propaquizafop was eliminated in the sterilised soil but was still accelerated with an increase in temperature, as the temperature accelerated the chemical degradation of propaquizafop in sterilised ginseng soil.

Based on the above results, an increase in soil moisture and sterilisation treatment can prolong the half-life of propaquizafop in soil, whereas an increase in culture temperature can shorten the half-life of propaquizafop in soil. Therefore, temperature had the most significant effect on propaquizafop degradation in the soil.

## Conclusions

In summary, we determined that propaquizafop was a highly adsorbed, less mobile pesticide in soils and was readily degradable in all five soils tested, indicating that propaquizafop is less likely to enter groundwater during its use and poses less environmental risk. In this study, the adsorption, migration, and degradation of propaquizafop in different soils were investigated. The results showed that the adsorption capacity of propaquizafop in different soils was strong, ranging from 46.98 to 57.76% at equilibrium, and the adsorption kinetics could be better fitted by the Elovich dynamics model (0.9882 < *R*^2^ < 0.9940). The Freundlich model can be used to better fit the adsorption characteristics of propaquizafop in different soils (0.9748 < *R*^2^ < 0.9885). Increasing the concentration of Ca^2+^ in the solution promoted the adsorption of propaquizafop in the soil. The mobility of propaquizafop in the soil was weak according to thin-layer chromatography and column leaching (0.076 < *R*_*f*_ < 0.123). The degradation rates of propaquizafop in the different soils were as follows: fluvo–aquic soil (half-life: 1.41 d), red loam (half-life: 2.76 d), paddy soil (half-life: 3.52 d), black soil (half-life: 5.73 d), and ginseng soil (half-life: 7.74 d). Environmental conditions such as temperature and soil water content, as well as microorganisms, are the main factors affecting the degradation of propaquizafop in the soil. Currently, there are no studies on the adsorption behaviour of propaquizafop in soil, and there are few studies on the effects of other environmental factors on propaquizafop in soil. These results will be useful for assessing the risk levels of propaquizafop in the environment.

## Data Availability

The datasets used and/or analysed in the current study are available from the corresponding author upon reasonable request.
